# Toward Autonomous Antibiotic Discovery

**DOI:** 10.1128/mSystems.00151-19

**Published:** 2019-06-11

**Authors:** Cesar de la Fuente-Nunez

**Affiliations:** a Machine Biology Group, Department of Psychiatry, Perelman School of Medicine, University of Pennsylvania, Philadelphia, Pennsylvania, USA; b Department of Microbiology, Perelman School of Medicine, University of Pennsylvania, Philadelphia, Pennsylvania, USA; c Department of Bioengineering, University of Pennsylvania, Philadelphia, Pennsylvania, USA

**Keywords:** computer-made drugs, antibiotic discovery, antimicrobial resistance, machines

## Abstract

Machines hold the potential to replace humans in many societal endeavors, and drug discovery is no exception. Antibiotic innovation has been stalled for decades, which has coincided with an alarming increase in multidrug-resistant bacteria. Since the beginning of the antibiotic era, the natural world has been our greatest innovator, giving rise to nearly all antibiotics available today. As mere observers of the vast molecular diversity produced by Earth’s organisms, we have perfected the art of isolating novel chemistries with life-saving antimicrobial properties. However, today we are at a crossroads, as no new molecular scaffolds have been discovered for decades. We may need to look beyond the natural world into the virtual dimension for solutions and harness present-day computational power to help solve the grand global health challenge of antibiotic resistance. Computer-made drugs may enable the discovery of unprecedented functions in biological systems and help replenish our arsenal of effective antibiotics.

## Abstract (precis)

Machines hold the potential to replace humans in many societal endeavors, and drug discovery is no exception. Antibiotic innovation has been stalled for decades, which has coincided with an alarming increase in multidrug-resistant bacteria.

## PERSPECTIVE

Nature has provided solutions to some of humanity’s most challenging problems, including the treatment of infectious diseases (1, 2). Minute organisms, such as bacteria and fungi, are the inventors (and producers) of antibiotics, arguably the greatest medicine ever invented. Penicillin, for example, the first antibiotic to be discovered, is produced by the fungus Penicillium chrysogenum (1). Since its discovery by Alexander Fleming in 1928, a wide range of other chemical structures have been isolated from the biological world. However, the rate of antibiotic discovery has decreased substantially since the 1980s (2–4). In the present day, the antibiotic development pipeline is essentially dry. What is the reason for this lack of innovation? Have we reached the limit of antibiotic discovery in the natural world? Perhaps Nature has by now shared all the secret molecules present in her recipe book, and it is time to look beyond. An alternative thought is that we may not be looking in the right places, or we may not have the right technologies needed to interrogate complex systems in the search for potent antibacterial drugs. Nevertheless, there is an urgent need to come up with out-of-the-box approaches to combat antibiotic resistance. What if we could teach computers the basic principles of chemistry, physics, and biology used by Nature to build antibiotics? What if we could then train machines to design such molecules by following Nature’s principles? Going a step further, could machines possibly create molecular structures unlike any that Nature has yet produced?

Here, I provide a brief overview of our recent efforts to develop computer-made antibiotics and speculate how machines might provide radical new ways of thinking about the drug discovery paradigm.

## THE ANTIBIOTIC RESISTANCE PROBLEM

Since the 1940s, when penicillin was first used, drug treatment has been able to cure many infections once considered lethal. The development of resistance, however, was observed from the very early days of antimicrobial discovery. In fact, in his Nobel lecture, Alexander Fleming stated, “It is not difficult to make microbes resistant to penicillin in the laboratory by exposing them to concentrations not sufficient to kill them, and the same thing has occasionally happened in the body” (5). We have learned that bacteria can readily become resistant to every antibiotic class that has made it into the clinic. From a bacterium’s point of view, this is not entirely surprising, as many of these miniscule organisms have the grand ability of replicating within minutes. This compressed time scale for propagation speeds up their evolution, thus favoring their capacity to overcome deleterious environments, such as a human body treated systemically with antibiotics, in order to ensure their own survival. Is it possible to design molecules that do not readily trigger resistance mechanisms in bacteria and perhaps molecules that bacteria have never encountered previously and against which cellular processes have not yet been developed?

In addition to bacteria’s own impressive ability to survive and adapt, other factors have contributed to the current global health crisis of antibiotic resistance. From a societal perspective, we have overused and misused life-saving antibiotic drugs, which has helped propagate resistance, via distinct mechanisms, from microbe to microbe. In fact, microbes are incredibly efficient at sharing and perpetuating pieces of DNA (i.e., plasmids) that contain genes that code for antibiotic resistance.

## THE END OF NATURE AS AN ANTIBIOTIC GENERATOR

Throughout history, Nature has been our greatest and most reliable ally for providing chemistries beneficial to human health. Specifically, most antibiotics have been produced in the natural world around us. However, in recent years, we seem to have reached a plateau in our ability to discover truly novel antimicrobial molecules. Exciting “new” molecules reported in the literature resemble past ones and operate via similar mechanisms of action that bacteria have encountered before, so bacteria may already have evolved resistance to them. Have we perhaps exhausted the vast source of therapeutic antimicrobial molecules available on Earth? The answer may be negative, and we may simply not yet have the technology needed to penetrate hidden sources of such drugs, or we may need to explore places where we have not traditionally ventured. Regardless, novel approaches are needed to discover new molecules that provide solutions to antibiotic resistance.

## COMPUTER-MADE ARTIFICIAL ANTIBIOTICS

**FIG 1:**
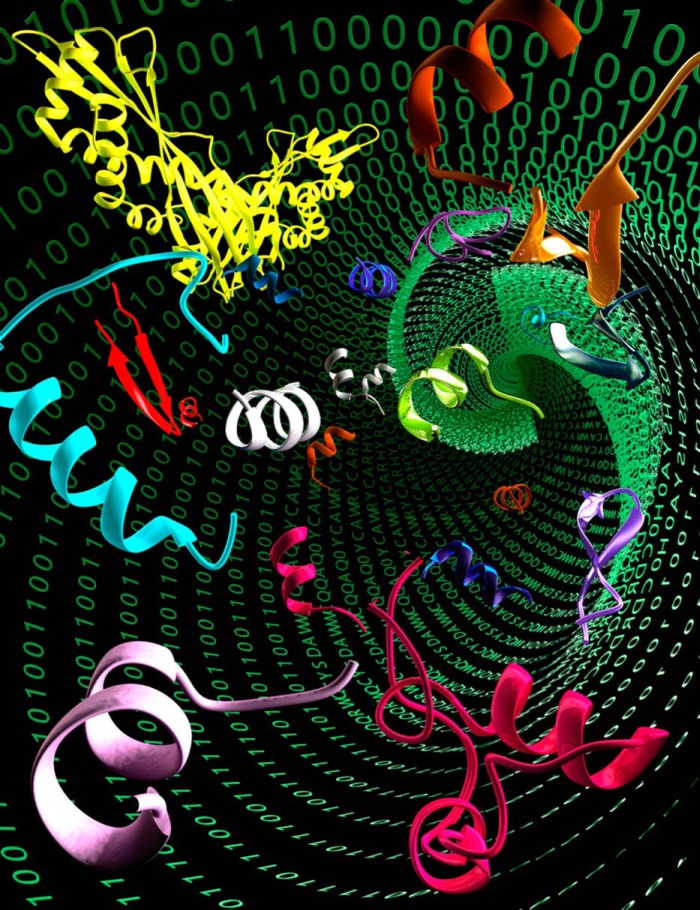
Computer-made artificial antibiotics. The future of antibiotic discovery may rely on virtual reconstructions and innovation based on existing biological systems. (Image courtesy of Ella Marushchenko, reproduced with permission.)

In light of the dire predictions for a future world without effective antibiotics, it is time to take drug discovery from the physical to the virtual domain (Fig. 1). Instead of relying exclusively on the beautiful structures produced in the biological world, can machines be “taught” the basic principles required to enable autonomous molecular discovery?

Proteins perform essentially every biological task required for life as we know it. Constituted of chains of amino acids, proteins are structural components of hair, skin, eyes, muscle, and other biological materials. They can also act as minimachines to catalyze key reactions that allow digestion, blood clotting, replication, transcription, and translation to occur. Proteins also contribute to metabolism, coordinate cellular events, modulate the immune system, and kill bacteria.

One extraordinary feature of proteins is their diversity. Proteins have nearly unlimited sequence space (or a possibility of sequence variants equal to 20*^n^*: there are 20 common amino acids, and *n* is the number of amino acids in a given protein chain). For this reason, proteins represent excellent scaffolds for engineering. To put this into perspective, the chemical combinatorial space of a peptide composed of just 25 amino acids (10^32^) surpasses the number of stars in the universe (10^31^). This likely means that hidden in this biological complexity, there is a world of previously unexplored sequences with unprecedented functions.

In fact, we have already discovered a family of tiny proteins called host defense peptides (HDPs) that are produced by all organisms on Earth and that are natural bacterial killers (2). For years, we have been dissecting and learning the basic chemical, physical, and biological principles that contribute to the antimicrobial properties of these HDPs (see, e.g., references [Bibr B6][Bibr B7][Bibr B10]).

Recently, we developed a genetic algorithm that enables machine-mediated evolution of HDPs to enhance their antimicrobial activity (11). Essentially, we trained a computer to execute Darwin’s evolutionary principles of mutation, selection, and recombination through a fitness function that selects for minimal structures capable of interacting with bacterial membranes (i.e., these minimal structures have a specific range of mean hydrophobicity and net charge). This approach, which built on the previously described *in vitro*-directed evolution of proteins (12) and previous molecular computational design strategies (see, e.g., references 13 and 14), has allowed exploration of sequence space and optimized HDPs to convert them into potent artificial antimicrobials that kill bacteria both *in vitro* and *in vivo*.

## PATTERN RECOGNITION-DRIVEN DISCOVERY

In addition to using computers to build novel antibiotics, can we browse through biological systems to find novel antimicrobial molecules? We recently described the use of pattern recognition-like systems to browse protein databases and, by decrypting embedded amino acid patterns, to identify antimicrobial peptides. This search was enabled by knowledge collected over decades on the molecular characteristics of HDPs, which are available in databases (see, e.g., reference 15). Such information allows biological information to be searched for patterns that resemble known HDPs. A protein database from the human body was explored, and hundreds of cryptic peptides were identified, a subset of which is produced in the human stomach (16). Because all computational approaches are predictions, we next validated these peptides by synthesizing them in Escherichia coli and collecting and purifying the end products. The molecules were tested for antimicrobial activity against a panel of foodborne pathogens (e.g., *Salmonella*) that naturally infect the human stomach. The cryptic peptides exhibited efficacy against such pathogens, displayed excellent safety profiles, and were active against bacteria in an animal model.

Similar approaches involving pattern recognition may play an important role in years to come to enable the exploration of previously unexplored biological systems and lead to the discovery of novel antibiotics.

## THE FUTURE OF ANTIBIOTIC DISCOVERY

Over the next 5 years, we aim to build the first class of machine-made artificial antibiotics to combat infectious diseases and antibiotic resistance and to discover novel antimicrobials in biological systems. The convergence of traditionally distinct disciplines, such as synthetic biology, chemistry, physics, and computer science, will be needed for this multidisciplinary endeavor. By investigating exciting questions in largely unexplored fields, we hope to gain ground-breaking insights, generate novel therapies, and outpace the ability of bacteria to evolve resistance mechanisms.
